# An Energy Efficient Adaptive Sampling Algorithm in a Sensor Network for Automated Water Quality Monitoring

**DOI:** 10.3390/s17112551

**Published:** 2017-11-05

**Authors:** Tongxin Shu, Min Xia, Jiahong Chen, Clarence de Silva

**Affiliations:** 1 Department of Electrical and Computer Engineering, University of British Columbia, Vancouver, BC V6T 1Z4, Canada; 2 Department of Mechanical Engineering, University of British Columbia, Vancouver, BC V6T 1Z4, Canada; minxia@mech.ubc.ca (M.X.); jhchen@mech.ubc.ca (J.C.); desilva@mech.ubc.ca (C.d.S.)

**Keywords:** adaptive sampling, water quality monitoring, power management, energy efficiency

## Abstract

Power management is crucial in the monitoring of a remote environment, especially when long-term monitoring is needed. Renewable energy sources such as solar and wind may be harvested to sustain a monitoring system. However, without proper power management, equipment within the monitoring system may become nonfunctional and, as a consequence, the data or events captured during the monitoring process will become inaccurate as well. This paper develops and applies a novel adaptive sampling algorithm for power management in the automated monitoring of the quality of water in an extensive and remote aquatic environment. Based on the data collected on line using sensor nodes, a data-driven adaptive sampling algorithm (DDASA) is developed for improving the power efficiency while ensuring the accuracy of sampled data. The developed algorithm is evaluated using two distinct key parameters, which are dissolved oxygen (DO) and turbidity. It is found that by dynamically changing the sampling frequency, the battery lifetime can be effectively prolonged while maintaining a required level of sampling accuracy. According to the simulation results, compared to a fixed sampling rate, approximately 30.66% of the battery energy can be saved for three months of continuous water quality monitoring. Using the same dataset to compare with a traditional adaptive sampling algorithm (ASA), while achieving around the same Normalized Mean Error (NME), DDASA is superior in saving 5.31% more battery energy.

## 1. Introduction

The present project of water quality monitoring has been primarily motivated by the fact that people in rural or undeveloped areas are at high risk of exposure to water-related diseases. However, the problem is not limited to such regions. Urban areas in industrialized countries are equally vulnerable. For example, recently, there were two major water-related crises that seriously affected Flint City, Michigan, USA, and White Rock, British Columbia, Canada. These problems could have been avoided and corrective actions could have been taken in a timely manner if a reliable, accurate, and distributed water monitoring system was available in the affected areas that could rapidly provide sufficient information about the water contamination.

Research on the subject of remote environmental monitoring has been prominent in recent years. One cannot overstate the importance of remote environmental monitoring, since it can result in convenience and flexibility of observing environmental conditions from a distance, thereby reducing the risks, cost, and the required time, while improving the accuracy and efficiency. Generally, in an automated water monitoring system, a wireless sensor network (WSN) is used [1,2], consisting of many sensor nodes that are capable of sensing data such as pH value, dissolved oxygen (DO), conductivity, oxidation-reduction potential (ORP), turbidity, temperature, and so on. The deployed sensors are typically able to sense, transmit and receive data in the wireless network [3]. Power management is of great significance not only in water quality monitoring but also in other types of remote environmental monitoring because of the importance of wisely managing the system power in order to sustain and extend proper operation of the system [4,5].

Ideally, the data sampling rate that is used for a sensor signal should depend on the rate at which the signal changes. The energy consumption for signal acquisition, processing, and transmission all depend on the sampling frequency, either directly or indirectly. Hence, for the sake of energy conservation, it is desirable to reduce the quantity of sampled data when the water quality remains relatively stable. Meanwhile, if certain parameters in the water are changing abruptly, the sampling frequency should be increased in order to acquire sufficient information about the condition of water. It should be noted that not only will the sampling frequency have a significant impact on the energy usage, the processing and transmitting of the sampled data will also consume extra energy. With this in mind, the proposed data-driven adaptive sampling algorithm (DDASA) has been developed, which dynamically changes the sampling frequency based on the nature of the sampled signal. This algorithm should be universally applicable with respect to conserving energy and prolonging the lifetime of the WSN. This approach is not limited to water quality monitoring but is applicable in other types of monitoring applications using WSN, such as indoor environmental monitoring [6], structural health monitoring [7], climate conditions monitoring [8], and healthcare monitoring [9].

The developed algorithm is implemented using MATLAB R2015a in the PC with a 3.20 GHz Intel Core i5-4460 CPU, 8 GB RAM and compared with a traditional adaptive sampling algorithm (ASA) [10]. The main contributions of this paper are as follows:

- A DDASA for energy conservation in a sensor network for automated water quality monitoring is presented.
- The universal applicability of the algorithm is validated with respect to various parameters with distinct characteristics, making it applicable in other types of practical monitoring situations.
- The proposed method is evaluated with respect to two key water-related parameters.
- The performance of DDASA is compared with the scheme of sampling at a fixed frequency in terms of data accuracy and energy conservation.
- The performance of DDASA is compared to a traditional ASA.

The rest of the paper is organized as follows: Section 2 outlines existing schemes for power management and some latest research that is directly related to this paper. In Section 3, the detailed mechanism of the proposed DDASA is presented, including how an abrupt environmental change is detected and how the sampling frequency is changed correspondingly. Section 4 demonstrates the experimental setup, and Section 5 provides some simulation results and a performance evaluation. Section 6 validates the proposed model. Finally, Section 7 concludes the paper.

## 2. Related Work

The subject of power management has been investigated by others in view of importance in various applications. In particular, the power management in WSN is a broad topic and can be studied based on various aspects. The general methodologies for managing power in a WSN can be briefly categorized as hardware and software design, network protocols and middleware services. The major energy-saving schemes designed under these four categories are mainly: radio optimization; battery repletion; sleep/wakeup schemes; energy-efficient routing, and; data reduction [11]. In terms of radio optimization, the traditional approaches focus on controlling the power used for signal transmission [12], in which the nodes in a WSN require knowing the power levels and link qualities of their neighbor nodes. Hence, the designed schemes have a spatiotemporal impact on the wireless sensor network, either locally or globally. With regard to battery repletion, in recent years applications have been broadly developed for remote environmental monitoring where the monitored environment is not easily accessible. Considering that it might be time- and manpower-consuming to replace the batteries, some work that focuses on the techniques of energy harvesting and wireless charging has been carried out [12]. Among the sleep/wake schemes, a typical one is duty cycling. This scheme is usually categorized as on-demand, asynchronous, and scheduled rendezvous [13]. In [5], a hybrid method based on the battery state and the stability of water quality has been proposed. The nodes can be switched on/off either depending on its remaining battery state or the need of sampled data. Moreover, in energy-efficient routing techniques, the distance between each regular node and also the sink node of the WSN plays a key factor. Hence, in order to optimally find a routing path that is energy efficient, schemes relying on single path and multiple paths have been proposed. A survey paper on this subject is available [14]. Data reduction exploits the fact that depending on the characteristics of the sampled data within the environment, some data could be redundant. For example, in water quality monitoring, if the monitored parameters remain relatively stable within the time period of interest, it is believed that no significant changes are happening. Thus, a relatively low sampling frequency may be used for the sake of saving energy. With fewer sampled data, the trend of the water quality can still be analyzed. This is the main advantage of using adaptive sampling for power management. Besides data reduction, there are some other similar approaches such as data aggregation and data compression [15] that also contribute to energy conservation by using fewer but representative data. Since the core algorithm proposed in this paper concerns adaptive sampling, a detailed review of the latest work in this topic is given next.

It can be desirable to predefine a standard for the quality of data when applying adaptive sampling techniques. Drira et al. [16] developed a location-aware scheme for an adaptive data collection system in vehicular networks, in which they constantly compare the travel time of a vehicle with a predefined value for deciding whether to transmit messages between a traffic management centre and a vehicle. It turns out that their developed algorithm is capable of reducing the communication load and data storage requirement while maintaining a high accuracy for estimating the fuel consumption and emission. Analogously, to ensure the quality of data, the proposed DDASA constantly compares the latest sampled data with a set of historical data to determine a new sampling frequency. However, the energy consumption in their methods is mainly reduced by limiting the number of transmission, while ours focuses on saving energy on the sensor node level.

Prabha et al. [17], on the other hand, proposed a context aware sensing technique which could be utilized for landslide monitoring. According to their approach, given a set of data, a discrete wavelet transformation is performed to find out the lowest sampling rate, which meanwhile should be able to guarantee the reliability of data. Based on the characteristics of data, three level thresholds are set to derive the sensor/network level contexts as Safe, Listen and Alert. The system initially starts to function at the lowest sampling rate until the sensor/network level contexts change (i.e., from Safe to Listen, or Listen to Alert). Hence, the sampling interval would be dynamically varied depending on the sensor/network-level contexts. As a result, the energy for sensory tasks could be saved. While their strategy is in a similar fashion to ours, they only consider three fixed sampling intervals (i.e., sampling frequencies), which need predefining manually. It should be believed that much more energy could possibly be saved if the scheme for changing sampling frequency could be data-driven, rather than three predefined sampling intervals.

In addition, an adaptive sampling approach for snow monitoring applications was developed in [10]. This method initializes the sampling rate by conducting a fast Fourier transform (FFT) on a sequence of pre-sensed data to acquire the maximum frequency, and depending on the subsequent data, a new maximum frequency is obtained. By comparing the variation of the current sampling rate and the new maximum frequency, a new sampling rate is made. However, this algorithm highly relies on a large number of pre-sensed data, and it also changes the sampling rate only depending on the FFT of the newly sampled data to avoid under-sampling. Nonetheless, in real environmental monitoring cases, under-sampling is acceptable if the frequency of the sensed data hardly fluctuates and remains relatively steady within a period of interest. In this manner, in order to dynamically change the sampling frequency, properly knowing the actual value differences and stability of the sensed data makes more sense than simply using an FFT on the subsequent data. This is essentially what makes DDASA different from a traditional ASA.

In [18], an adaptive sampling scheme for acquiring wind data is proposed based on energy awareness. Rather than being data accuracy oriented, this algorithm is essentially energy awareness oriented. This means when the remaining energy of the battery drops below a predetermined threshold, the sampling frequency will be decreased accordingly. The larger the gap between the remaining battery state and the predetermined threshold, the faster the drop of the sampling frequency as a result of maximizing the lifetime of the battery. Apparently, much more energy could be saved when the battery state is relatively low through this proposed scheme. However, since there is always a tradeoff between the accuracy of the sensed data and the energy conservation, a strategy that is primarily driven by the battery state will surely compromise the accuracy of the sensed data, and possibly even under-sampling can occur as a result. Hence, it is believed that the scheme DDASA proposed in this paper, which is data accuracy driven, would be more reasonable and universally applicable.

Moreover, it has to be pointed out that, despite that transmitting and receiving data wirelessly also consume an enormous amount of energy, the energy consumption of sensing is not always insignificant [19]. In particular, some energy hungry sensors, for instance, the gas sensors, also consume a large amount of energy compared to that used in data transmission [10,20,21,22,23,24]. The work presented in this paper focuses on saving energy from the sensory task within each single node in the WSN, which is typically beneficial for using energy-hungry sensors.

## 3. Data-Driven Adaptive Sampling Algorithm

The core idea behind the DDASA is to design a data accuracy-driven strategy of power utilization in water quality monitoring using autonomous sensor nodes, depending on the environmental changes. It is clear that a higher sampling frequency is desired where the water quality is vulnerable to rapid environmental change or pollution, particularly in a post-industrial city. Particularly, the researchers and water quality observers of such areas might be more concerned and sensitive to the sudden changes of certain key parameters in the water. Correspondingly, however, if the monitored parameters hardly fluctuate, meaning no significant changes are taking place, it is believed a lower sampling frequency is preferred, and as a result, less energy will be needed for data sampling, data processing and transmission.

Based on these assumptions, a revised sigmoid function is proposed in the present DDASA. This function is used to dynamically change the sampling frequency, which is expressed as:

(1) $$y(D)=\frac{2}{1+e^{-(D-t)}}$$

(2) $$D=\frac{\left|X_{i+1}-X_{i}\right|}{\frac{1}{N}\sum_{i-N+1}^{i}X_{i}}$$

**Figure 1:**
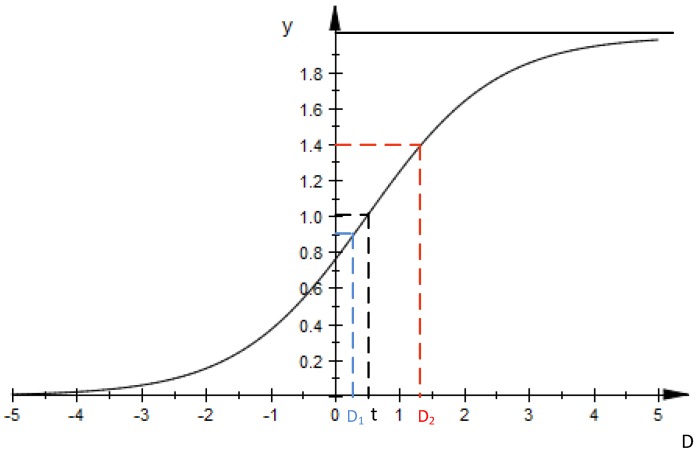
Representation of the revised sigmoid function.

Here, *t* is a pre-determined threshold; and *D* is the absolute difference between *X_i+_*_1_ and *X_i_* over the average value of a number of *N* sliding-window based most recent data. Since we are interested in knowing whether there exists a sudden environmental change or not, it is reasonable to compare the latest sensed data *X_i+_*_1_ with the former data *X_i_* in the signal sequence, and then divide the absolute difference between with the mean value of the most recent *N* data. If *D* is sufficiently large, it indicates a sudden environmental change. Then a somewhat higher sampling frequency is desired. Additionally, if the value of *D* is smaller than the threshold *t*, which means the value changes are not significant enough, the sampling frequency can be reduced in view of the relative stability of the monitored data. Hence, the theoretical value of *y* is actually smaller than 2 but greater than *y*(0), that is, since the smallest value of *D* will not be a number smaller than 0 in data sensing process, the value of *y*(0) is essentially greater than 0 regardless of the value of *t*. A representation of the revised sigmoid function is presented in Figure 1. It is found the value of *y* in the simulation is mostly a number either slightly smaller than 1 (e.g., when *D* equals to *D*_1_) when the sensed data numerically remain stable or greater than 1 (e.g., when *D* equals to *D*_2_) when the sensed data abruptly change.

Since the value of *y*(*D*) dynamically changes depending on the latest sampled data, the sampling frequency should also be changed accordingly. If the current sampling frequency is denoted by *f_curr_*, which is used to acquire latest data, then the new sampling frequency, denoted by *f_new_* is represented as:

(3) $$f_{new}=f_{curr}\times y\left(D\right)$$

Hence, a new sampling frequency for the next iteration is a function of the newly sampled data and the average value of the latest *N* sliding window-based data, which satisfy the needs for a reasonable manner of sampling as well as the needs for energy conservation.

In summary, a pseudo code for implementing the DDASA is represented in Algorithm 1 as below:

**Algorithm 1. DDASA**1.Initialize a constant sampling frequency denoted as
$f_{const}$, sample a number of *N* for later use; store the samples in a sequence as S;2.Predetermine a threshold t according to the characteristics of the monitored parameter;3.Define
$D=\frac{\left|X_{i+1}-X_{i}\right|}{\frac{1}{N}\sum_{i-N+1}^{i}X_{i}}$; 4.Define
$f_{curr}$ = $f_{const}$;5.**for** (*i* = *N*; *i*++) {6. Sample $X_{i+1}$ based on $f_{curr}$ (or $f_{curr}{}^{\prime }$);7. $D=\frac{\left|X_{i+1}-X_{i}\right|}{\frac{1}{N}\sum_{i-N+1}^{i}X_{i}}$;8. $y(D)=\frac{2}{1+e^{-(D-t)}}$ ;9. $f_{new}=f_{curr}\times y\left(D\right)$;10. $f_{curr}{}^{\prime }=f_{new}$; 11. $S\left(i+1\right)=X_{i+1}$;}12.**end**13.**return**
*S*;

It should be noted that the use of a sigmoid function corresponds with both the physical nature of the sampling process and the sensed data. This scheme ensures that a new sampling frequency to be adjusted not just based on the latest sensed data, but also a set of past-period data which reflect the overall environmental conditions throughout the time. More precisely, a faulty reading, whose value might be unusually higher or lower than the average, would not change the next sampling frequency drastically. Instead, if an abrupt change is detected, which constantly leads certain reading numerically increase or decrease, the future sampling frequency will therefore increase gradually based on a consecutive set of obtained data. In equivalent words, a much higher sampling frequency is the consequence of using *f_curr_* to multiply *y*(*D*) (greater than 1 but lower than 2) in each iteration for multiple times after comparing the latest acquired data with a set of past-period data. As a result, this data-driven scheme allows the energy to be reasonably either consumed or conserved, as it is the trend of the sensed data rather than certain unusually high or low value data that decides future sampling frequency.

## 4. Illustrative Simulation

The performance of the proposed algorithm is assessed by using a set of real-time data provided by National Oceanic and Atmospheric Administration (NOAA) [25]. NOAA uses distributive platforms (buoys) to form a network for collecting real-time water-quality data. The data chosen for the simulation are collected from a platform called “Jamestown,” where the monitoring duration ranges from 15 December 2016 to 15 March 2017 with a sampling interval of 1 h for a sample. To validate the robustness of the DDASA, two distinct parameters, turbidity and DO, are selected since their value range differs enormously among various water-related parameters.

In order to evaluate the performance for different values of the pre-determined parameters, the term Normalized Mean Error (NME) is introduced, which indicates the overall goodness of fit and is defined as:

(4) $$NME=\frac{1}{n}\sum_{i=1}^{n}\left|{\hat{x}}_{i}-x_{i}\right|\times 100\%$$

Here, ${\hat{x}}_{i}$ denotes the normalized *i*th data in the reconstructed signal, $x_{i}$ represents the normalized *i*th data in the original signal, and *n* denotes the total number of data in the reconstructed data set. In general, different parameters correspond with different numeric ranges. For example, the measured pH values typically lie in the range from 0 to 14, while the sensed value of conductivity in water can reach as high as 500 μS/cm. Based on NME, the overall goodness of fit can be directly indicated regardless of the numeric scale of sensed data.

## 5. Simulation Results

The simulation results are divided into two parts. First, the proposed DDASA is tested using DO and turbidity data separately, followed by the selection of a list of parameters and the corresponding performance indicator (i.e., NME). Later, with regard to energy conservation, a comparison between the proposed algorithm of the present paper and a traditional adaptive sampling is presented. The simulation results demonstrate that the DDASA algorithm is not only capable of maintaining a high level for energy-efficient sampling, but also effectively reconstructing the original signal with much less number of data.

**Figure 2:**
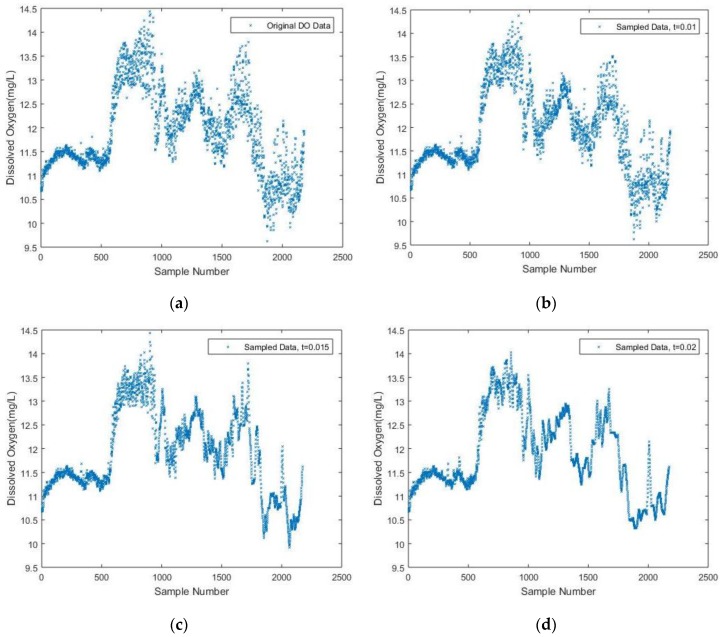
(**a**) Original dissolved oxygen (DO) Data; (**b**) Sampled DO data with *t* = 0.01; (**c**) Sampled DO data with *t* = 0.015; (**d**) Sampled DO data with *t* = 0.02.

First, a plot of the original DO data signal is shown in Figure 2a for later comparison, which consists of 2182 sensed samples in total. To interpret the original signal, it can be inferred that an abrupt change in the water might occur around the 600th sample, leading to a significant increase of the DO content.

In the simulation, the initial sampling frequency is set to the same value as the constant frequency used for sampling the 2182 data. The window size (i.e., *N*) is set to 50, while the threshold is a variable. Then a linear interpolation between two neighboring measurements is used to fit the reconstructed signal with respect to different threshold values. Hence, the trend of the DO parameter is given intuitively.

In Figure 2b–d, the simulation results with different threshold values are presented. It is seen that as the predetermined threshold increases, fewer sample data are acquired based on the DDASA. Combined with the linear interpolation fitting, the trend of the DO content can still be maintained at a high level of similarity compared to the original DO signal when *t* = 0.01, *t* = 0.015 and *t* = 0.02, respectively.

**Figure 3:**
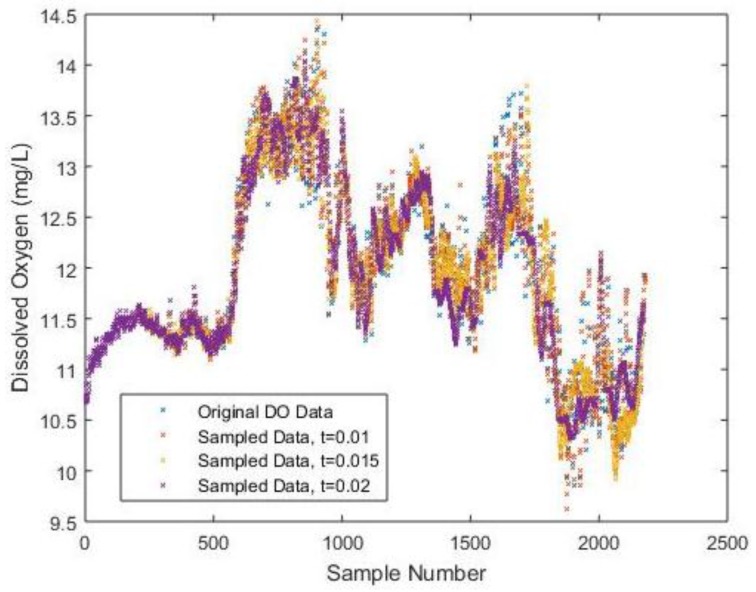
The difference among DO data for different sampling intervals.

To provide a more intuitive understanding when adopting different sampling intervals, the difference among the sampled DO data is shown in Figure 3 correspondingly, which is the outcome of a combination of Figure 2a–d.

**Figure 4:**
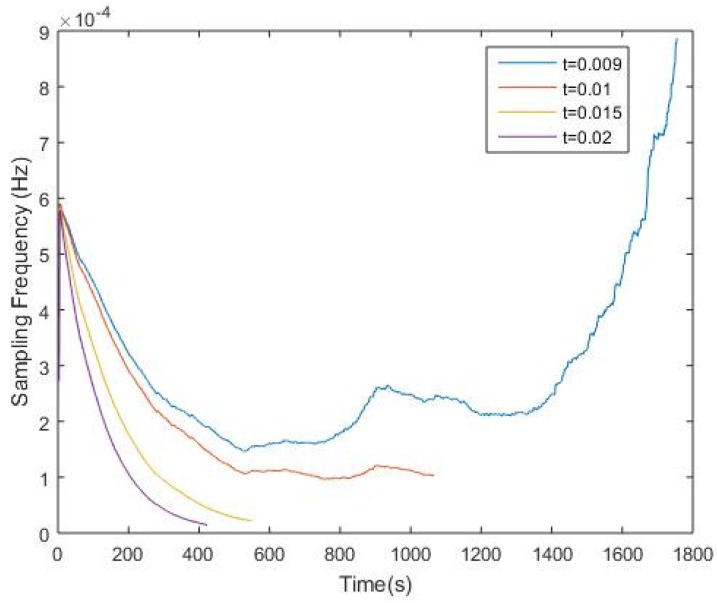
Frequency trend with *t* = 0.009, *t* = 0.01, *t* = 0.015and *t* = 0.02, respectively.

The frequency trend for each corresponding threshold is also shown in Figure 4. It is believed that when the threshold is set to a proper number, (i.e., *t* = 0.01), the sampling frequency will eventually converge or remain at a stable level. However, it is also found out that when the threshold is set to a value smaller than 0.01, for instance, when *t* = 0.009, the frequency trend will not converge. This happens mainly because the value of *D*(*i*) in most iterations is greater than the threshold, which results in a constant increase in the sampling frequency. This also means the dynamically changed sampling frequency is not sensitive to the data change, which in fact is not a desired outcome. Additionally, when *t* = 0.015 and *t* = 0.02, compared to the case when *t* = 0.01, the sampling frequency is more sensitive to the data change, and as a consequence, more energy will be conserved since the sampling frequency in each iteration is lower than the initial sampling frequency.

**Figure 5:**
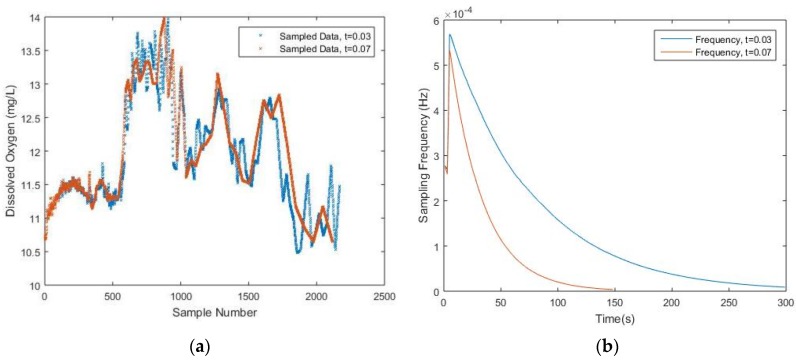
(**a**) Sampled DO data with *t* = 0.03 and *t* = 0.07; (**b**) Frequency trend with *t* = 0.03 and *t* = 0.07.

In Table 1, an investigation of the algorithm performance and the predetermined threshold values is given. It is noticed that although the value of the predetermined threshold increases evenly, the number of samples drops unevenly, however, the NME increases as the number of samples decreases. In Figure 5, it is obvious that some important peaks are missing due to a lack of sampled data where the threshold equals to 0.03 and 0.07, respectively. The corresponding NME are 9.99% and 11.70%, which is relatively high. Thus, in order to find a suitable value for the threshold, it would be necessary to preset a minimum required data quality in terms of NME and, meanwhile, the threshold value should not be too small, which ensures that the sampling frequency will eventually converge. More details regarding the choice of threshold value will be discussed later.

**Figure 6:**
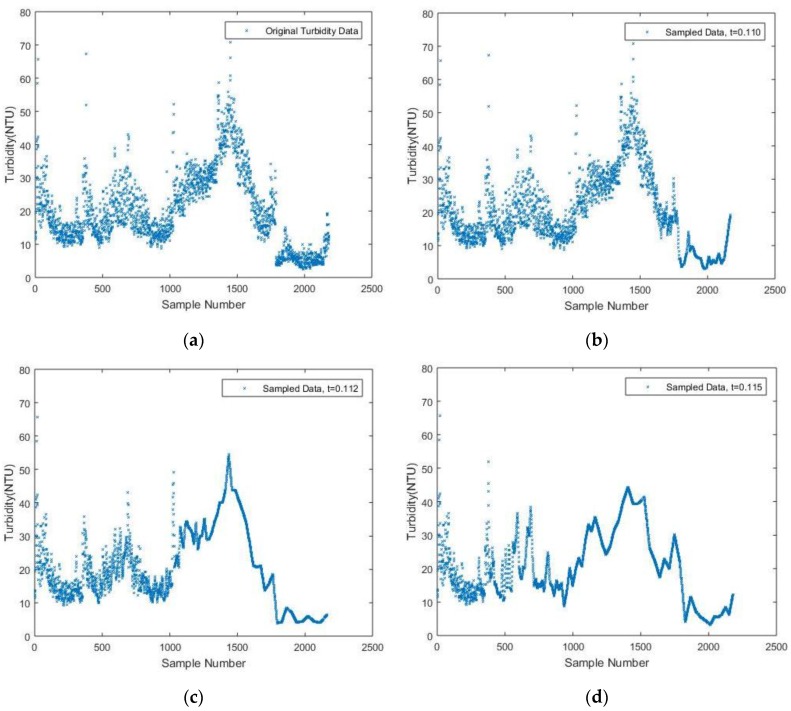
(**a**) Original Turbidity data; (**b**) Sampled Turbidity data with *t* = 0.110; (**c**) Sampled Turbidity data with *t* = 0.112; (**d**) Sampled Turbidity data with *t* = 0.115.

A similar simulation is conducted based on turbidity data. The results are presented in Figure 6 with threshold values of 0.110, 0.112 and 0.115. A comprehensive comparison is found in Table 2. The same linear interpolation scheme is implemented between two neighboring measurements to fit the plot.

It still can be shown that when the threshold value increases from 0.110 to 0.112, the number of samples drastically drops from 1591 to 422, while a 4.26% NME is still tolerable when t equals 0.112. As *t* increases from 0.112, the number of samples decreases correspondingly, which is, however, not as many as the case when t increases from 0.110. Thus, it is reasonable to believe that setting the threshold value as 0.112 is relatively a suitable choice, while choosing a value of 0.115, 0.120 or 0.140 for *t* is also acceptable considering their corresponding NME. Since only 422 or less data values are sampled with the DDASA, which is less than quarter the original data values, it is believed that the energy consumed for data acquisition, storage and transmission will be significantly reduced. Thus, the lifetime of the battery will be prolonged while maintaining a high level of accuracy within the sampled data compared to the original ones.

**Figure 7:**
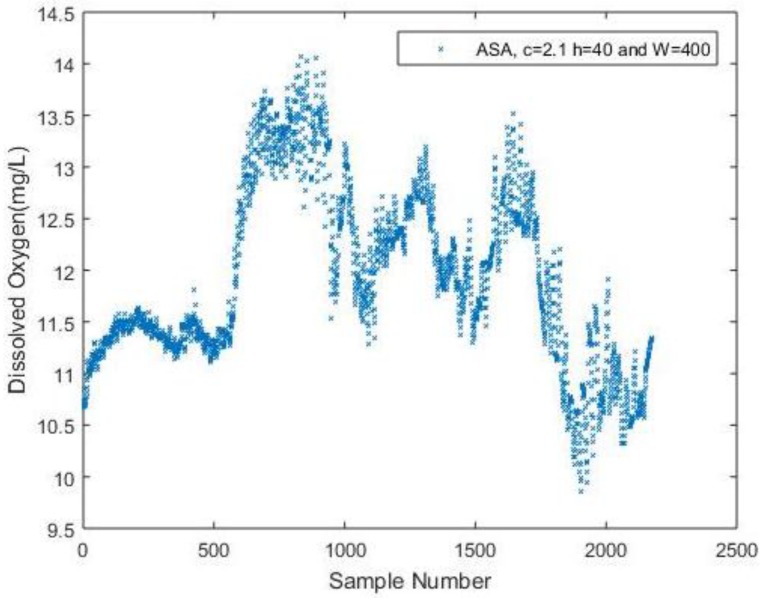
Reconstructed DO signal based on adaptive sampling algorithm (ASA).

**Figure 8:**
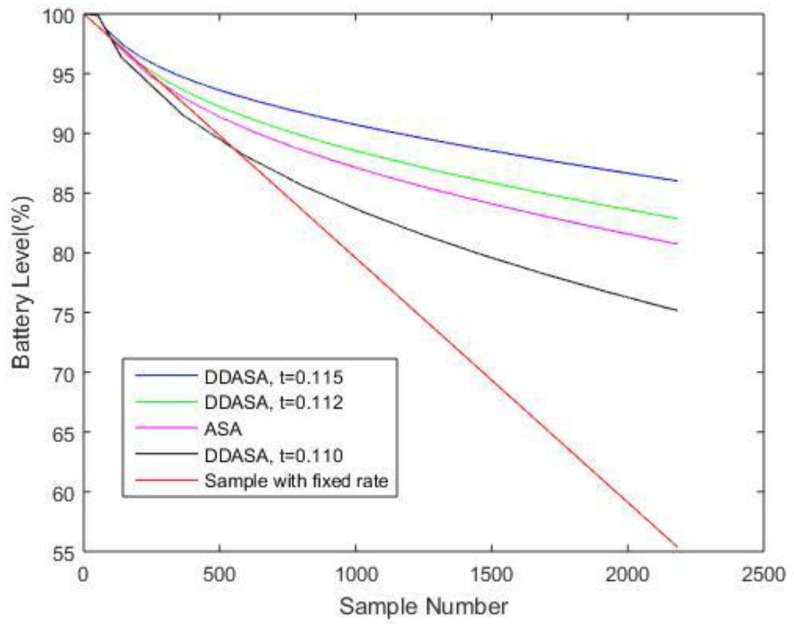
Algorithm performance with respect to the remaining battery level.

A comparison of algorithm performance is presented in Figure 7 and Figure 8, and Table 3. Using the same sequence of DO data, a traditional ASA is implemented as well. A set of parameter values are chosen as empirically suggested in paper [10]. Figure 7 shows the reconstructed signal using ASA can still indicate the fluctuation of the DO compared to the original signal in Figure 2a. The NME for the results is 5.31% with a number of 637 samples. The performance is similar when *t* = 0.115 for analyzing DO data in the proposed DDASA. However, to achieve a similar result, the ASA uses 637 samples in total, which proves to be more energy-consuming in contrast to the proposed DDASA, where only a number of 320 is needed. Figure 8 presents the comparison of energy consumption over time for ASA and for different values of predetermined thresholds of DDASA. Considering the fact that a more frequent sampling process will equivalently increase the energy consumption for storing and transmitting data, to simplify the comparison process, only the energy consumed for keeping a node active and for data acquisition is considered. Moreover, the active time of the node in each case will be equivalent, which means it should be equal to the total length of time for a fixed rate sampling process. The specific number of energy consumption for different sensing activities can be found in [20], which is the basis for obtaining the simulation results in Figure 8. In Table 3, a performance comparison is provided, in which the DDASA outperforms the ASA when *t* = 0.115 and *t* = 0.112 with respect to the remaining battery level.

## 6. Model Validation

In the developed scheme above, finding an appropriate value for the threshold plays a key role in determining the performance of the proposed algorithm. Considering that the value of *D* in Equation (2) is highly related to not only the absolute value between the newest sample data and its former one, but also the mean value of a set of window-based data. Thus, the function for looking for a suitable threshold value should be in a similar way, which is defined as:

(5) $$t=\frac{\frac{1}{k-1}\overset{k}{\underset{i=2}{\sum }}\left|x_{i}-x_{i-1}\right|}{\frac{1}{k}\overset{k}{\underset{i=1}{\sum }}x_{i}}=\frac{k}{k-1}\cdot \frac{\overset{k}{\underset{i=2}{\sum }}\left|x_{i}-x_{i-1}\right|}{\overset{k}{\underset{i=1}{\sum }}x_{i}}$$

To validate the proposed model, a k-fold cross-validation has been conducted based on Equation (5). To simplify the cross-validation process, a 5five-fold cross-validation has been selected in regard to the total number of data. Meanwhile, the whole set of data has been divided into five equal sized subsets, four of which are utilized as training sets while the remaining subset functions as a testing set. In each cross-validation process, a threshold value will be given depending on Equation (5), in which *X_i_* is the sensed data amongst the training set. Afterwards, based on the very threshold value, a NME will be obtained comparing the reconstructed signal against the training set. If each of the five subsets is denoted as A, B, C, D and E separately, the outcome for the cross-validation is presented in Table 4.

It turns out that through the cross-validation process, the average value of NME is 2.93% while a suggested average value of threshold is 0.01172. This threshold value and its corresponding NME also correspond with the results presented in Table 1, where a slightly smaller value of *t* results in a smaller NME, while a slightly larger value of *t* leads to a higher NME.

**Figure 9:**
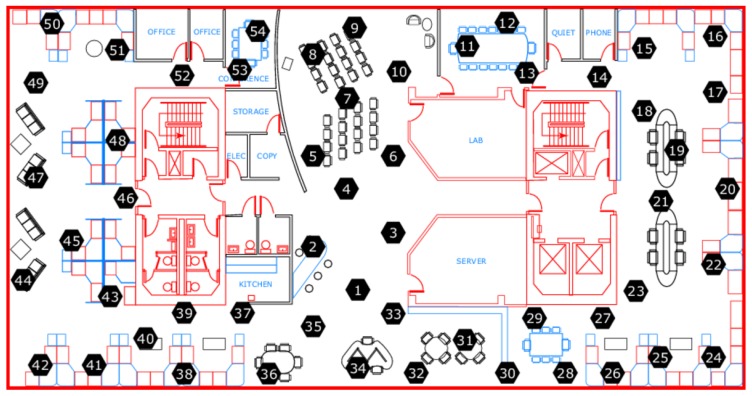
Wireless sensor network (WSN) setup in the Intel Berkeley Research Lab.

**Figure 10:**
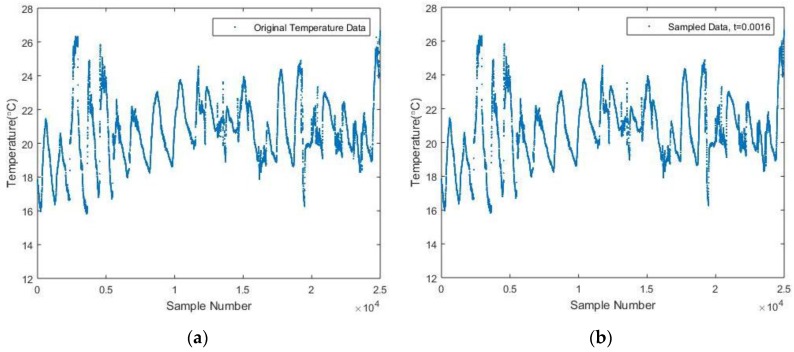
(**a**) Original Temperature data from Intel Berkeley Research Lab; (**b**) Sampled Temperature data with 0.0016.

Also, to prove that this algorithm is universally applicable, a different dataset from Intel Berkeley Research lab [26] has been utilized. The dataset is comprised of real-time data for temperature, humidity, light and voltage measured by Mica2Dot sensor nodes within a WSN (see Figure 9). In particular, a set of temperature data consisting of 25,000 samples, which were collected by Sensor 13 from 28 February 2004 to 20 March 2004, is selected as the original data set. Based on the whole data set, a suggested threshold for sampling is derived based on Equation (5), which is 0.0016. Afterwards, the reconstructed signal is given in Figure 10, with an NME equal to 0.08% and 17,957 samples needed. Since around 7000 samples are deducted comparing with the original data set, it should therefore be believed much energy can be saved throughout the sampling process. Furthermore, since the accuracy of the reconstructed signal is 99.2% when *t* equals to 0.0016, for the sake of saving more energy, slightly increasing the value of *t* and thus reducing the sample numbers should also be acceptable. For instance, when *t* increases to 0.0020, the corresponding NME is 0.29%, while only 11,423 samples are necessarily collected.

## 7. Conclusions

In this paper, a data-driven adaptive sampling algorithm (DDASA) for node-level sampling was presented. It was proven that this algorithm was robust for different types of parameter sampling, and could effectively conserve energy with a satisfactory reconstructed signal. Compared with some existing adaptive sampling algorithms, which are battery-state driven, there are justifiable occasions where the sampling frequency is based on the real-time sampled data, especially when the fluctuation of the environmental parameters, is of significant interest. Additionally, it was shown that, by dynamically changing the sampling frequency according to the newly sampled data, the proposed DDASA would outperform a traditional ASA with respect to the accuracy of the reconstructed signal and the energy conservation. Thus, the goal of prolonging the life time of the nodes has been achieved with the proposed approach.

Possible future work will involve considering a hybrid approach based on DDASA, in which the priority can be given either to data accuracy or battery state based on the real-time level of data fluctuation and the percentage of remaining battery energy. Since there exists a tradeoff between the data accuracy and battery state, it is speculated that a hybrid method will maintain a good accuracy of data samples while maximizing the lifetime of the sensor nodes.

## Figures and Tables

**Table 1:** Results with different threshold values for DO data sampling.

	*t* = 0.01	*t* = 0.015	*t* = 0.02	*t* = 0.03	*t* = 0.07
Number of Samples	1064	548	421	297	146
Normalized Mean Error (NME)	1.62%	5.52%	8.43%	9.99%	11.70%

**Table 2:** Turbidity data sampling results for different threshold values.

	*t* = 0.110	*t* = 0.112	*t* = 0.115	*t* = 0.120	*t* = 0.140
Number of Samples	1591	422	320	244	172
NME	1.14%	4.26%	5.33%	5.39%	6.16%

**Table 3:** Performance comparison between ASA and data-driven adaptive sampling algorithm (DDASA) using DO data.

	DDASA (*t* = 0.115)	DDASA (*t* = 0.112)	ASA	DDASA (*t* = 0.110)	Fixed Rate Sampling (*f* = 1/3600 Hz)
Number of Samples	320	422	637	1591	2182
NME	5.33%	4.26%	5.31%	1.14%	0
Remaining Battery Level	86.03%	82.86%	80.72%	75.19%	55.37%

**Table 4:** Model validation using five-fold cross-validation.

Training Sets	ABCD	ABCE	ABED	ACED	BCED
Testing set	E	D	C	B	A
Threshold	0.0102	0.0113	0.0115	0.0120	0.0136
NME	3.37%	3.40%	3.12%	2.53%	2.25%
